# Volatile Distribution in Flowers of *Lathyrus odoratus* L. by HS-SPME-GC Technique and Enantiomeric Separation Data

**DOI:** 10.3390/plants13233272

**Published:** 2024-11-21

**Authors:** James Calva, Mayerly Parra, Ángel Benítez

**Affiliations:** 1 Departamento de Química, Universidad Técnica Particular de Loja, San Cayetano alto s/n, Loja 1101608, Ecuador; 2 Carrera de Bioquímica y Farmacia, Universidad Técnica Particular de Loja, San Cayetano alto s/n, Loja 1101608, Ecuador; mdparra2@utpl.edu.ec; 3 Biodiversidad de Ecosistemas Tropicales-BIETROP, Herbario HUTPL, Departamento de Ciencias Biológicas y Agropecuarias, Universidad Técnica Particular de Loja (UTPL), San Cayetano s/n, Loja 1101608, Ecuador; arbenitez@utpl.edu.ec

**Keywords:** *Lathyrus odoratus*, SPME, α-trans-bergamotene, enantiomeric distribution, heatmap

## Abstract

*Lathyrus odoratus* L., commonly known as sweet pea, is a plant with a distinctive aroma that can develop in various habitats. An analysis of the aromatic profile of the species was conducted using the HS-SPME (solid-phase microextraction headspace) technique. This study aimed to explore the composition of and variation in the floral scent emissions of *L. odorathus*. The floral scents from fresh flowers were collected over different months and analyzed using gas chromatography coupled with mass spectrometry on apolar and polar stationary phase columns. In the apolar column, the majority compounds included linalool (19.27–5.79%), α-trans-bergamotene (29.4–14.21%), and phenyl ethyl alcohol (30.01–1.56%), while on the polar column, the predominant compounds included myrcene (13.25%), (E,E)-α-farnesene (26.33–8.16%), α-trans-bergamotene (42.09–24.82%), and others. This investigation was complemented by enantioselective analysis using a chiral phase based in cyclodextrins, which revealed the presence of (1*R*)-(+)-α-pinene, (*S*)-(−)-limonene, (*R*)-(+)-germacrene D, and (R)-(E)-nerolidol as enantiomerically pure components and linalool as a racemic mixture. Notably, the principal component analysis (PCA) and heatmap revealed variations among the chemical compounds collected at different harvest times. This demonstrates that temporal factors indeed impact chemical compound production. Furthermore, research on the aromatic properties of flowers provides a theoretical basis for studying and improving the components of their scent.

## 1. Introduction

Aromatic plants, also known as herbs and spices, have been used for their preservative and medicinal properties since around 5000 BC [1]. Different cultures have been extracting unusual aromas, a source of great fascination, by means of techniques that have become popular over the years. The application of these aromas in as the food, cosmetics, and pharmaceutical industries is particularly noteworthy [2].

The Fabaceae family has 770 genera and 19,500 species thanks to its wide distribution, making it part of the third-largest plant family in the world [3]. Its chemical composition includes mainly terpenes (mono and sesquiterpenes), fatty acids, and benzenoids [4]. Due to the large family and the variety of compounds, it has diverse properties for the treatment of ailments, pathologies, and syndromes [5].

The genus *Lathyrus* comprises 160 species [6] that have a remarkable ability to tolerate hostile environmental conditions such as drought, bogs, and low temperatures. Approximately twenty tree species are endemic of South América [7]. *Lathyrus odoratus* L., known as the sweet pea, is an annual climbing plant native to southern Europe and north Africa [8]. It is prized for its showy and fragrant flowers, which are very delicate to harvest and have a very short lifespan. In Ecuador, *Lathyrus odoratus* L. is an introduced species and can be considered a herb or vine. It inhabits the coastal or Andean region, can grow up to 3000 m above sea level, and can be found in the provinces of Guayas and Pichincha [9]. In this study, this species was identified for the first time in the south of Ecuador. According to the IUCN (International Union for Conservation Union) Red List of Threatened Species, the species is currently threatened with extinction [10].

Various data on the volatile chemistry of this species are already available. Previous studies have reported the chemical composition of *Lathyrus odoratus* L. Essential oil, it consists of (E)-β-ocimene (22.9–46.5%) linalool (16.6–26.2%), geraniol (4.5–6.5%), nerol (3.3–10.1%), α-trans-bergamotene (1.3–6.8%), and β-sesquiphellandrene (0.2–1.2%) as the main compounds [11]. This plant has been used as an antidiuretic and to supply calcium to the body [12]. In other species, germacrene D (50.4%), germacrene B (18.7%), γ-elemene (9.5%), and myrcene (7.4%) are the main components in the oil of *Lathyrus rotundifolius* [13]. Additionally, involatile compounds isolated in *L. odoratus* were an unidentified carbohydrate L-1-*O*-methyl-*myo*-inositol and L-bornesitol [14]. Other volatile compounds in *Lathyrus* L. species were analyzed by SPME-GC-MS, and the main components of *L. aphaca* were tetradecane 14.3%, camphor 21.6–10.1%, and *yomogi* alcohol 26.1–16.5%; those of *L. cicera* camphor 18.7–2.0% and yomogi alcohol 20.3–3.0%; those of *L. gorgonei* yomogi camphor 17.1–9.0% and alcohol 24.5–13.1%; those of *L. sativus* camphor 9.0% and yomogi alcohol 11.4%; those of *L. ochrus* hexenal 7.0% and 2-methyl butanoic acid 7.2%; those of *L. saxatilis* tetradecane 5.4%, (Z)-3-hexenal 6.4%, and hexanal 7.7%; and those of *L. blepharicarpos* var. *cyprius* dodecane 5.1%, yomogi alcohol 5.9%, and (Z)-3-hexenal 8.6% [15].

In this study, the main aromatic compounds present in *Lathyrus odoratus* L. were studied using the HS-SPME technique with the aim of discovering the components determining its fragrance, as well as taking advantage of the applicability of the technique of headspace solid-phase microextraction coupled with a gas chromatograph (HS-SPME-GC) for the extraction and analysis of volatile compounds and determining the compounds present at different times of collection. In the same way, we studied the enantiomeric composition of the volatile fraction and report it for the first time. We describe and apply the technique of enantioselective gas chromatography (GC) to assign the absolute configuration of chiral natural compounds. This configuration strongly influences the odor properties of their enantiomers [16,17]. Thus, for the first time, the present study focused on the variability of the chemical profile of Ecuadorian flowers in different months of the year to determine the best period for harvesting and achieving the highest level of desirable bioactive compounds for use in the pharmaceutical and food industries.

## 2. Results

### 2.1. Chemical Composition

**Figure 1:**
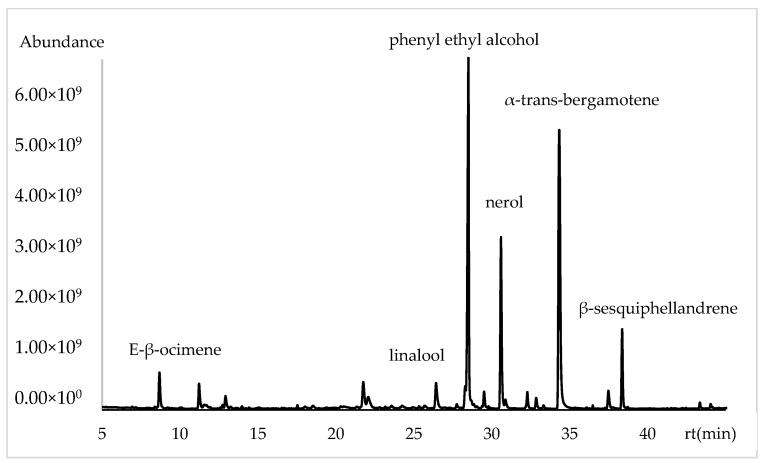
Chromatogram of volatile chemical composition of *Lathyrus odoratus* flowers in apolar column.

The variability in the chemical composition of the volatile compounds from the flowers of *Lathyrus odoratus* L., analyzed using the DB5-ms column and ordered by retention index, is presented in Table 1. The major constituents in *L. odoratus* flowers were α-trans-bergamotene (14.21%, 29.35% and 28.68%), phenyl ethyl alcohol (24.67%, 7.58% and 7.16%), nerol (16.18%, 2.22% and 5.61%), linalool (5.79%, 18.6% and 19.27%), myrcene (3.48%, 1.41% and 1.20%), β-sesquiphellandrene (2.76%, 4.95% and 7.56%), and 2-phenyl ethyl acetate (2.35%, 0.35% and 0.38%) (Figure 1).

**Figure 2:**
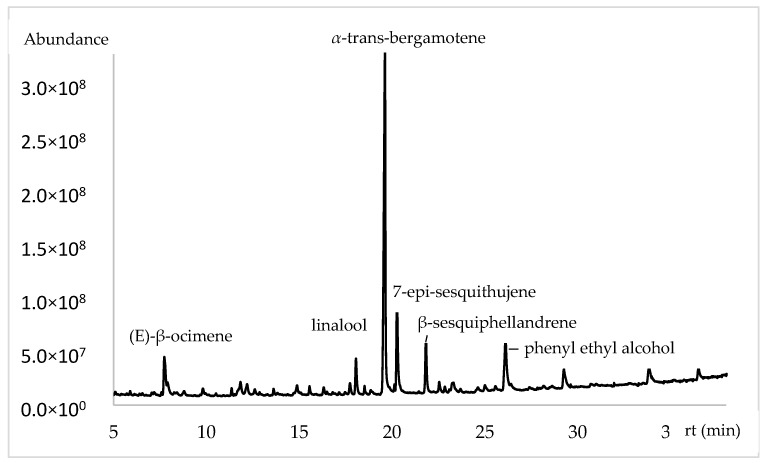
Chromatogram of volatile chemical composition of *Lathyrus odoratus* flowers in polar column.

The chemical composition of flowers using a polar phase based on polyethylene glycol (PEG) (Table 2 and Figure 2) showed variability in the chemical volatile majority compounds present during the studied months. The principal compounds were α-trans-bergamotene (42.09%, 43.48% and 24.82%), (E)-β-ocimene (12.14%, 7.21% and 2.89%), linalool (8.94%, 9.38% and 2.88%), *β*-sesquiphellandrene (7.03%, 6.53% and 0.33%), 7-epi-sesquithujene (5.21%, 4.30% and 2.03%), phenyl ethyl alcohol (8.16%, 1.76% and 30.01%) and finally (E)-nerolidol (1.43%, 1.21% and 5.30%).

### 2.2. Enantiomeric Analysis

**Figure 3:**
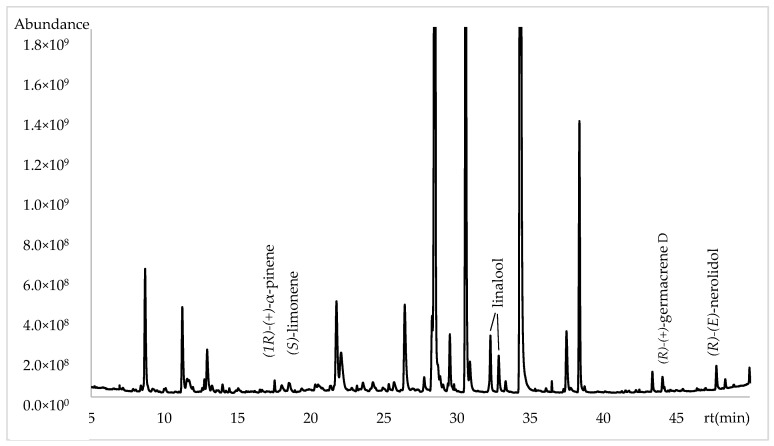
Chromatogram of enantioselective analysis in *Lathyrus odoratus* flowers using β-cyclodextrin column.

The enantioselective analysis was performed with a capillary column using 2,3-diethyl-6-tert-butyldimethylsilyl-β-cyclodextrin as a chiral selector. A total of six enantiomers were identified, along with their respective enantiomeric distribution and enantiomeric excess (*e.e.*). The enantiomers were (1*R*)-(+)-α-pinene, (*S*)-(−)-limonene, (*R*)-(+)-germacrene D and (*R*)-(E)-nerolidol were identified as enantiomerically pure components and the linalool as a racemic mixture. The complete enantioselective analysis is presented in Table 3 and Figure 3.

### 2.3. Statistical Analysis

The principal component analysis (PCA), corresponding to the DB-5ms column, showed that component 1 explained 66.81% of the variance, while component 2 had 33.19% of the variance in the total analysis (Table 4).

**Figure 4:**
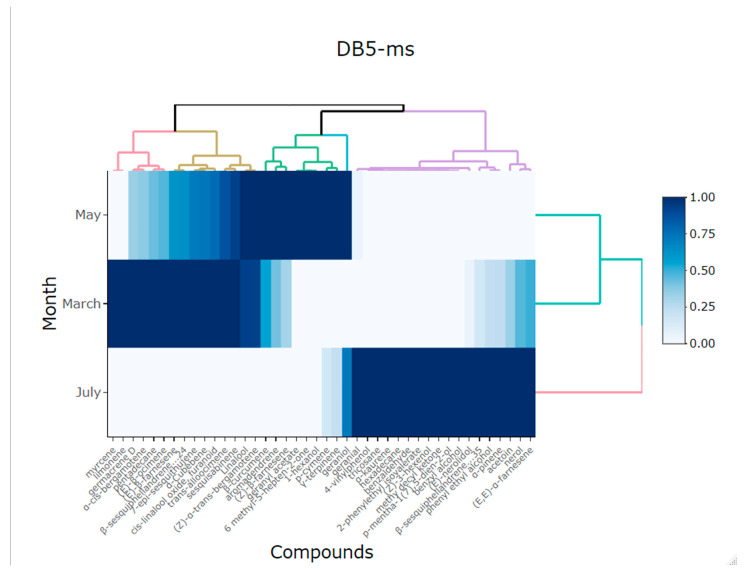
Heatmap showing the main compounds of *Lathyrus odorarus* L. in different months of harvest using DB5-ms column.

The heatmap in Figure 4 shows two separate groups of the volatile compounds from the flowers of *Lathyrus odoratus* L. for DB5-ms column in relation to month. In this heatmap, the scale of color is relative to the value of the volatile compounds. The high similarity among volatile compounds in March and May is indicated by the dominance of the blue. On the other hand, where the similarity between compounds is lower, the similarity (e.g., July) is indicated in light blue. The results obtained on the basis of the tool used were able to better group all the compounds, thus identifying those that stand out more in the different months of collection, being the months of April and May, that present a greater variation in compounds. It should be emphasized that the compounds that predominate more in the month of May are α-trans-bergamotene, (E)-β-ocimene, and (Z)-β-farnesene and in April are nerol and phenyl ethyl alcohol.

**Figure 5:**
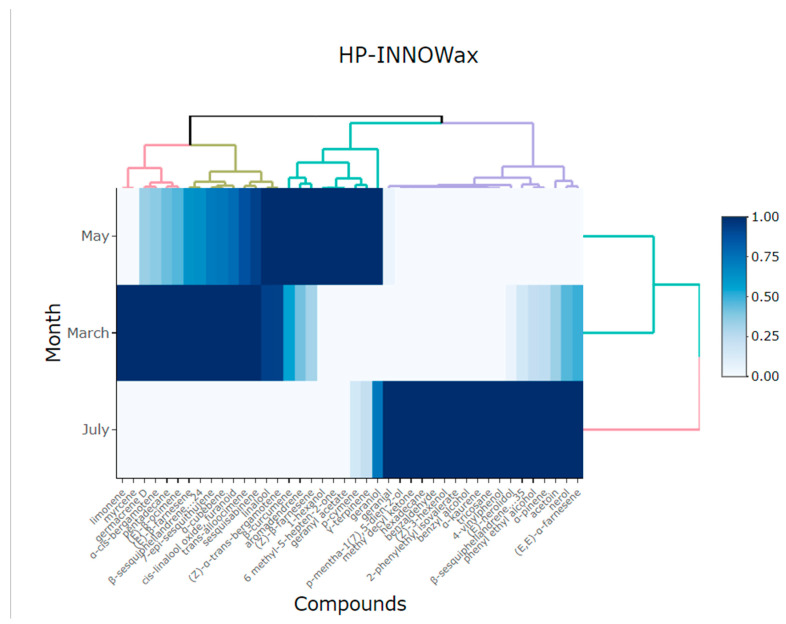
Heatmap showing the main compounds of *Lathyrus odorarus* L. in different months of harvest using a HP-INNOWax column.

PCA showed the variability between the two components corresponding to method 2 (HP-INNOWax): component 1 had a percentage of 75.97% and component 2 had 24.02%, giving a total of 99.99% of the explained variance (Table 4). The heatmap in Figure 5 shows two separate groups of volatile compounds from the flowers of *Lathyrus odoratus* L. for the HP-INNOWax column in relation to month. The high similarity among volatile compounds in March and May is indicated by the dominance of the blue in the heatmap. It was possible to identify the components that stood out in the different months of collection, with June being the month with the greatest variability of compounds, but it should be noted that the most predominant compounds were phenyl ethyl alcohol, geranial, α-trans-bergamotene, (Z)-β-farnesene, and (E,E)-α-farnesene.

## 3. Discussion

Volatile compounds emitted from fresh *Lathyrus odoratus* flowers were obtained using HS-SPME-GC analysis on a DB-5MS column and 71 compounds were isolated. The analysis showed that the main volatile compounds in quantifiable amounts (>2%) were α-trans-bergamotene, (E)-β-ocimene, linalool, (E,E)-α-farnesene, 7-epi-sesquithujene and β-sesquiphellandrene. Volatile profiles similar to those we have recorded for flowering *L. odoratus* L. have been reported, such as (E)-β-ocimene, linalool, (E,E)-α-farnesene and β-sesquiphellandrene as the predominant volatiles [39]. Porter (1999) [11] studied the floral volatiles of *L. odoratus* L. using thermal desorption–gas chromatography–mass spectrometry and the major components were (E)-β-ocimene (22.9–46.5%), linalool (16.6–26.2%), geraniol (4.5–6.5%), and nerol (3.3–10.1%), and in lower proportions α-trans-bergamotene (1.3–6.8%) and β-sesquiphellandrene (0.2–1.2%). In another study of *L. odorathus* flowers in four locations in the United Kingdom, the most abundant compounds were consistently found to be (E)-β-ocimene (22.9%, 27.8%, 35.3% and 46.5%) and linalool (16.6%, 20.7%, 23.6% and 26.2%) [11]. Bruce et al. (2002) evaluated the insecticidal properties of the species and identified three primary compounds responsible for this activity: linalool, phenylacetaldehyde, and benzyl alcohol [40]. Other studies on volatile compounds identified in different species, for example, HS-SPME of *Lathyrus vernus* L. collected in Turkey identified three major compounds: 1-octen-3-ol (49.8%), 2-hexenal (9.9%), and linalool (3.8%) [41]. A rather different composition was described for the volatile fraction of *L. rotundifolius* essential oil collected in Iran, and five major compounds were found: germacrene D (50.4%), germacrene B (18.7%), γ-elemene (9.5%), myrcene (7.4%), and β-sesquiphellandrene (2.6%) [13]. All of these studies demonstrate the variability in chemical composition between the same species collected in different locations, as well as between different species of the same genus. The major compound in our study was α-trans-bergamotene, which is used in applications in cosmetology and perfumery. In cosmetics, it is widely used due to its ability to refresh and flavor products, while in perfumery, it contributes a characteristic citrus aroma appreciated for its freshness and vitality, and this component exhibits antioxidant, anti-inflammatory, and antimicrobial properties, making it a beneficial ingredient for skin and hair care [42].

According to Sexton et al. (2005) [43], the synthesis of the floral aroma of the species *L. odoratus* L. develops in parts of the flower such as the standard petals and wings. The authors showed that the production of the characteristic aroma is due to the condensation of vapor, which contains an abundant quantity of terpenes surrounding the flowers. The increase in the emission of volatile compounds occurs in the final stages of flower opening, with the best sampling time being when the flowers are fully open.

The chemical composition of plants is subject to both quantitative and qualitative variations. Plant material collected at different times of the year may contain novel compounds with distinct bioactivities [44]. Seasonal variations in chemical composition may be influenced by phenological status and environmental conditions, which regulate biosynthesis [45]. Additionally, the location where the species is collected, including factors such as plant care, climatic conditions, soil nutrients, and pollinators, can differ from the geographical areas referenced in other studies. There is also limited information on the resulting compounds based on different collection times [46]. Research has extensively investigated the effect of seasonal changes on the production of secondary metabolites in plants, revealing variations in specific compounds produced during different seasons. These variations in phytochemical production significantly alter the chemical profile of plant materials, potentially affecting the quality of bioactive compounds. In addition, seasonal variations in chemical composition can influence the biological activity of the plant [47]. The flowers of *L. odoratus* L. contain anthocyanins with antioxidant, antiulcer and anti-inflammatory activities. Four major anthocyanins were identified in dark-pink flowers and the components of an alcoholic extract were analyzed. The total anthocyanins showed higher antimicrobial activity than the isolated compounds, being more effective against bacteria, yeasts and fungi [48].

Regarding the HS-SPME-GC analysis, Lancioni et al. (2022) mentioned that the SPME technique has many advantages, one of the most important being the non-incorporation of solvents to obtain the compounds during the desorption phase, which helps reduce environmental pollution caused by the solvents used [49].

A limitation of this research is the results obtained by GC-MS analysis in both columns, which reported different peak area percentages for the same compound. These results could be related to the following factors. (1) It is important to consider the effect of the column used on the selectivity of the separation, which may lead to variability in the elution of the compounds and co-elution of the peaks. (2) Different columns have different stationary phases, which affect the retention times of the compounds and thus the separation of the analytes [50]. (3) Columns with different polarities, such as apolar and polar, may separate structurally similar compounds differently, resulting in variations in the chromatographic profile [51]. (4) Take into account that for the polar stationary phase, the samples were stored. According to some studies, storage for one day can affect the emission of volatile compounds responsible for the aroma [52]. The synthesis and continuous emission of these compounds is interrupted due to lack of access to water and nutrients from the parent plant [53], and can lead to a reduction in the intensity of the aroma and changes in the composition of the emitted volatile compounds. Therefore, the GC-MS results in our research are limited to the description of the compounds obtained by DB5-ms and HP-INNOWax.

To the best of our knowledge, this is the first enantioselective analysis of *Lathyrus odoratus* L. flowers. This understanding is crucial, as it allows us to determine their significance, which varies depending on the analyte being studied. Accurate knowledge of the enantiomeric ratios of aroma compounds is becoming increasingly important, particularly in the authentication of food products and essential oils, as well as in the development and creation of fragrances and perfumes [54]. Chiral discrimination is recognized as one of the key principles in biological activity and olfaction [55].

## 4. Materials and Methods

### 4.1. Plant Material

**Figure 6:**
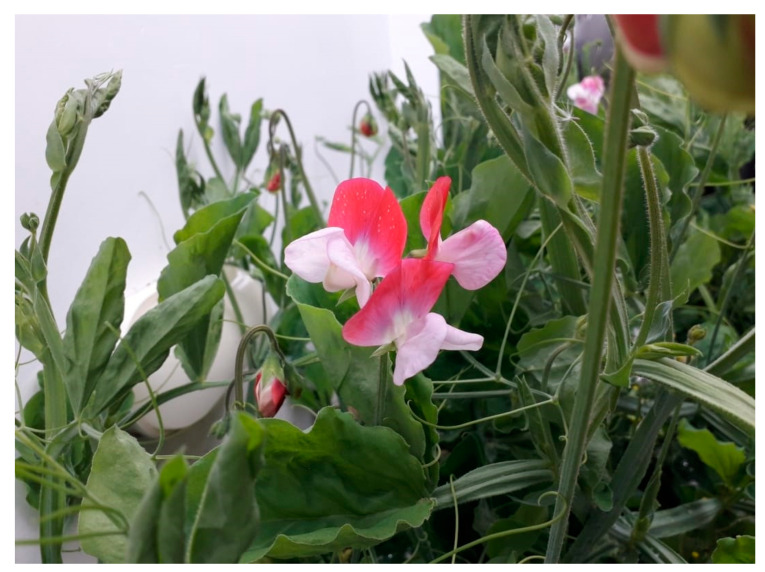
*Lathyrus odoratus* L. in the flowering period.

*Lathyrus odoratus* L. flowers (Figure 6) were harvested in the morning, its were analyzed immediately in the apolar stationary phase and stored for 24 h for the polar stationary phase, from March to July 2023 in the Quisquinchir district, Saraguro Canton, Loja Province, at coordinates of 3°36′43.8″ S; 79°14′43.2″ W at an altitude of 2600 m a.s.l. A voucher specimen (14777) has been deposited in the HUTPL herbarium. This collection was carried out with the authorization of the Ministry of Environment, Water and Ecological Transition MAATE-ARSFC-2022. Our study was based on a short period of observation, less than 6 months, and it was always difficult to extrapolate and generalize these results, as Ecuador produces little seasonality throughout the year. There are only two defined seasons: wet or winter (October to May), and dry or summer (June to September). In our study, samples were collected in both seasons. The mean annual temperature is 12–13 °C with relatively little monthly variation (data from the local meteorological unit of INAMHI). The annual precipitation at the INAMHI station M142 in Saraguro (79°23′ W 3°62′ S 2525 m a.s.l.) is 827 mm, calculated from the last 25 years [56]. The soils of this area were formed on granodioritic plutonic rocks, partially sheared, and metamorphosed [57].

### 4.2. Extraction of Compounds by SPME

The SPME device and the fused silica fibers were purchased from Supelco (Bellafont, PA, USA). The fibers based in divinylbenzene/carboxene/polydimethylsiloxane (DVB/CAR/PDMS) (model 57328-U, Supelco, Bellefonte, PA, USA) of 10 mm length and conditioned prior to use at 270 °C for 1. The analytical conditions described for HS-SPME sampling were chosen after preliminary assays using different amounts of flowers and different extraction and desorption conditions (time, temperature, equilibrium time). Cut samples (5 g) were placed separately in glass vials (100 mL) and sealed hermetically using PTFE/silicone septa. The flowers were left at 40 °C with agitation (250 rpm) for 10 min to allow equilibration of volatiles in the headspace. After equilibration, the SPME needle was inserted and the fiber was exposed to the headspace for 40 min. The volatiles adsorbed were thermally desorbed in the hot injection port of a GC for 5 min at 250 °C with the purge valve off in splitless mode and deposited onto a capillary column [58].

### 4.3. Chemical Profiling

#### 4.3.1. GC-MS

Analytical gas chromatography was carried out using a Thermo Scientific model TRACE 1310 chromatograph (Waltham, MA, USA) equipped with a Thermo Scientific model ISQ 7000 mass spectrometer (Bartlesville, OK, USA) to analyze the volatile compounds. The carrier gas used was ultrapure helium (GC purity grade from Indura, Guayaquil, Ecuador) with a flow rate of 1 mL/min [59]. It was performed in splitless mode, with an injector temperature of 250 °C. The separation was achieved using two columns: a DB-5ms fused silica column (5% phenyl 95% polydimethylsiloxane, 30 m × 0.25 mm i.d., film thickness 0.25 µm) and an HP-INNOWax (polyethylene glycol, 30 m × 0.25 mm i.d., film thickness 0.25 µm) from J&W Scientific (Folsom, CA, USA). The column temperature was 40 °C for 5 min with a ramp of 3 °C/min to 150 °C, a second ramp of 5 °C/min to 180 °C, a third ramp of 7 °C/min to 230 °C, and finally held for 10 min. The ionization source and quadrupole temperatures were 230 °C and 150 °C, respectively, with a run time of 67 min. The spectra recordings represented a full scan with a mass range (30 and 350 amu) at a scan rate of 0.2 scan/s [60].

The identification and determination of the constituents of each profile was tentatively made by comparing their mass spectral fragmentation patterns and linear retention indices (LRIs) relative to C9–C24 n-alkanes (Sigma-Aldrich, St. Louis, MO, USA, EE.UU.) with those reported in the literature, as well as those stored in an MS spectral literature database (NIST 2020), and an acceptable difference from literature data was ±25 units in LRI.

#### 4.3.2. Enantioselective Analysis

The enantioselective analysis was carried out using an enantioselective MEGA-DEX-DAC Beta from Mega, MI, Italy, capillary column based on 2,3-diethyl-6-tert-butyldimethylsilyl-β-cyclodextrin. The column was 25 m long, 0.25 mm in internal diameter and with 0.25 μm phase thickness, installed in the same GC–MS instrument described for the qualitative analysis. Sample volumes, injector temperature, transfer line temperature, and MS parameters were the same as for the qualitative analyses, whereas the split ratio was 20:1. The GC method was as follows. Initial temperature was 60 °C for 2 min, followed by 2 °C/min to 220 °C, which was maintained for 2 min. A homologous series of n-alkanes (C9–C25) was also injected in order to calculate the linear retention indices. The enantiomers of the chiral components were identified by injection of enantiomerically pure standards (Sigma-Aldrich, St. Louis, MO, USA) [61].

### 4.4. Statistical Analysis

Principal component analysis (PCA) was used to comprehend the similarity among the volatile compounds of essential oils in relation to the months for DB5-ms columns and HP-INNOWax [62]. A heatmap was generated using pheatmap and ggplot2 packages to visualize the similarity of chemical compositions of the volatile compounds from the flowers of *Lathyrus odoratus* L., using the DB5-ms column and HP-INNOWax ordered by retention time. Before conducting the heatmap analysis, the data were standardized to a common scale (0 to 1), where color intensity was used to represent the effect size. These results were obtained with the statistical software Rstudio version 1.1.453 [63].

## 5. Conclusions

In this study, the combination of GC-SPME-MS and chemometric analysis provided a robust and reproducible method for the analysis of *Lathyrus odoratus* L. flowers. By integrating both polar and apolar phases in gas chromatography (GC), we identified a wide range of aromatic compounds. This study is the first to report the emission of chiral compounds from *L. odoratus* L. flowers, thereby improving our understanding of their chemical profile. Significant variations in chemical composition were observed at different flower harvests, leading to the identification of specific volatile compounds. In addition, the season of harvest was found to influence the chemical composition of *L. odoratus*. The observed seasonal variations provide valuable insights for selecting the optimal season to harvest components of interest, thereby enhancing the potential applications of *L. odoratus* L. in the food and pharmaceutical industries.

## Figures and Tables

**Table 1:** Volatile chemical composition of *Lathyrus odoratus* flowers in apolar column in different collection periods (2023).

DB-5ms							
N°	Compound	LRI ^a^	LRI ^b^	March (% ± SD)	May (% ± SD)	July (% ± SD)	MF
1	methyl hexanote	930	921 [18]	n.d.	0.08 ± 0.14	n.d.	C_7_H_14_O_2_
2	α-pinene	933	932 [18]	0.15 ± 0.01	0.35 ± 0.02	0.40 ± 0.01	C_10_H_16_
3	octen-3-ol	987	974 [18]	0.46 ± 0.03	0.26 ± 0.01	n.d.	C_8_H_16_O
4	myrcene	991	988 [18]	3.48 ± 1.2	1.41 ± 0.3	1.20 ± 0.2	C_10_H_18_
5	cis-pinane	1000	982 [18]	0.82 ± 0.4	n.d.	n.d.	C_10_H_18_
6	ethyl-(3E)-hexenoate	1003	1003 [18]	0.67 ± 0.03	0.24 ± 0.01	n.d.	C_8_H_16_O_2_
7	limonene	1031	1024 [18]	0.29 ± 0.03	0.46 ± 0.05	n.d.	C_10_H_16_
8	benzene acetaldehyde	1032	1036 [18]	0.58 ± 0.02	0.73 ± 0.11	1.1 ± 0.03	C_8_H_8_O
9	(E)-β-ocimene	1048	1044 [18]	1.48 ± 0.2	4.62 ± 0.7	4.23 ± 0.61	C_10_H_16_
10	(2E)-octen-1-al	1051	1049 [18]	0.26 ± 0.01	0.35 ± 0.02	0.36 ± 0.01	C_8_H_14_O
11	trans-arbusculone	1074	1066 [18]	n.d.	0.68 ± 0.11	0.30 ± 0.02	C_9_H_14_O_2_
12	n-octanol	1077	1063 [18]	0.20 ± 0.34	0.11 ± 0.18	n.d.	C_8_H_18_O
13	trans-linalool oxide (furanoid)	1090	1084 [18]	0.32 ± 0.02	0.28 ± 0.01	0.39 ± 0.01	C_10_H_18_O_2_
14	camphenilone	1097	1078 [18]	n.d.	n.d.	0.49 ± 0.01	C_10_H_18_O
15	ethyl heptanoate	1100	1097 [18]	0.36 ± 0.5	n.d.	n.d.	C_9_H_18_O_2_
16	linalool	1103	1095 [18]	5.79 ± 0.7	18.6 ± 0.9	19.27 ± 0.63	C_10_H_18_O
17	cis-rose oxide	1112	1106 [18]	1.85 ± 0.23	1.4 ± 0.11	n.d.	C_10_H_18_O
18	phenyl ethyl alcohol	1123	1107 [18]	24.67 ± 0.56	7.58 ± 0.76	7.16 ± 0.96	C_8_H_10_O
19	β-pinene oxide	1155	1154 [18]	n.d.	0.84 ± 0.11	0.35 ± 0.01	C_10_H_16_O
20	citronellal	1157	1148 [18]	0.11 ± 0.19	n.d.	n.d.	C_10_H_18_O
21	benzene acetic acid	1184	1175 [18]	0.12 ± 0.21	n.d.	n.d.	C_9_H_10_O_2_
22	neoisomenthol	1195	1184 [18]	0.14 ± 0.25	n.d.	n.d.	C_12_H_26_
23	methyl salicylate	1201	1190 [18]	n.d.	0.12 ± 0.20	n.d.	C_8_H_8_O_3_
24	nerol	1227	1227 [18]	16.18 ± 0.9	2.22 ± 0.13	5.61 ± 0.27	C_10_H_18_O
25	citronellol	1231	1223 [18]	0.08 ± 0.14	n.d.	0.13 ± 0.21	C_10_H_20_O
26	cis-carveol	1234	1226 [18]	0.37 ± 0.07	n.d.	1.38 ± 0.12	C_10_H_16_O
27	(2E)-decenal	1245	1260 [18]	n.d.	n.d.	0.67 ± 0.03	C_11_H_22_O
28	neral	1245	1235 [18]	1.58 ± 0.04	n.d.	n.d.	C_10_H_16_O
29	geraniol	1255	1249 [18]	2.12 ± 0.16	0.49 ± 0.01	0.39 ± 0.01	C_10_H_18_O
30	methyl citronellate	1258	1257 [18]	n.d.	0.09 ± 0.15	n.d.	C_11_H_20_O_2_
31	2-phenyl ethyl acetate	1263	1254 [18]	2.35 ± 0.23	0.35 ± 0.01	0.38 ± 0.01	C_10_H_12_O_2_
32	decanol	1266	1266 [18]	0.68 ± 0.05	n.d.	n.d.	C_10_H_22_O_2_
33	geranial	1275	1264 [18]	1.26 ± 0.13	0.68 ± 0.10	n.d.	C_10_H_16_O
34	2-undecanone	1294	1293 [18]	0.8 ± 0.02	0.56 ± 0.01	0.65 ± 0.03	C_11_H_22_O
35	tridecane	1309	1300 [18]	0.34 ± 0.06	n.d.	0.22 ± 0.02	C_13_H_28_
36	cis-2,3-pinanediol	1319	1318 [18]	n.d.	n.d.	0.27 ± 0.01	C_8_H_9_NO_2_
37	2-methyl-4-methylhexyl ester butanoic acid	1322	1304 [18]	n.d.	n.d.	0.02 ± 0.03	C_15_H_24_
38	methyl geranate	1324	1322 [18]	0.39 ± 0.04	n.d.	0.21 ± 0.01	C_11_H_18_O_2_
39	silphinene	1337	1345 [18]	n.d.	n.d.	1.39 ± 0.12	C_12_H_24_O
40	α-cubebene	1345	1348 [18]	0.46 ± 0.01	0.85 ± 0.02	0.39 ± 0.01	C_15_H_24_
41	methyl-anthranilate	1348	1334 [18]	1.38 ± 0.12	1.89 ± 0.16	0.78 ± 0.09	C_8_H_9_NO_2_
42	n-undecanol	1380	1367 [18]	n.d.	n.d.	0.07 ± 0.12	C_15_H_24_
43	7-epi-sesquithujene	1384	1390 [18]	1.2 ± 0.27	3.56 ± 0.71	4.06 ± 0.63	C_15_H_24_
44	methyl decyl ketone	1386	1388 [18]	0.18 ± 0.01	0.38 ± 0.02	0.57 ± 0.01	C_12_H_24_O
45	β-cubebene	1388	1387 [18]	0.4 ± 0.01	0.58 ± 0.03	n.d.	C_15_H_24_
46	n-tetradecane	1394	1400 [18]	0.95 ± 0.02	n.d.	n.d.	C_14_H_30_
47	α-funebrene	1404	1402 [18]	n.d.	n.d.	0.46 ± 0.07	C_15_H_24_
48	(Z)-caryophyllene	1411	1408 [18]	0.26 ± 0.01	0.78 ± 0.01	0.24 ± 0.01	C_15_H_24_
49	α-trans-bergamotene	1431	1432 [18]	14.21 ± 0.37	29.35 ± 1.18	28.68 ± 1.38	C_15_H_24_
50	(Z)-β-farnesene	1440	1440 [18]	1.73 ± 0.2	4.72 ± 0.7	5.64 ± 0.9	C_15_H_24_
51	(E)-β-farnesene	1452	1454 [18]	1.36 ± 0.06	0.98 ± 0.08	2.04 ± 0.17	C_15_H_24_
52	sesquisabinene	1457	1457 [18]	0.36 ± 0.01	0.56 ± 0.03	0.42 ± 0.01	C_15_H_24_
53	(2E)-docecenal	1472	1464 [18]	n.d.	n.d.	0.79 ± 0.12	C_15_H_24_
54	n-dodecanol	1475	1469 [18]	1.37 ± 0.04	n.d.	0.04 ± 0.06	C_12_H_26_O
55	γ-gurjunene	1476	1477 [18]	n.d.	0.65 ± 0.01	n.d.	C_15_H_24_
56	germacrene D	1478	1480 [18]	0.85 ± 0.02	0.97 ± 0.09	0.58 ± 0.04	C_15_H_24_
57	ar-curcumene	1481	1479 [18]	0.03 ± 0.05	0.58 ± 0.02	0.08 ± 0.15	C_15_H_22_
58	α-zingiberene	1491	1493 [18]	n.d.	n.d.	0.13 ± 0.22	C_15_H_26_O
59	pentadecane	1492	1500 [18]	n.d.	0.64 ± 0.04	n.d.	C_15_H_32_
60	(E,E)-α-farnesene	1499	1505 [18]	0.78 ± 0.02	0.56 ± 0.01	0.59 ± 0.01	C_15_H_24_
61	β-curcumene	1509	1514 [18]	n.d.	0.68 ± 0.02	0.81 ± 0.01	C_16_H_32_
62	β-sesquiphellandrene	1523	1521 [18]	2.76 ± 0.8	4.95 ± 1.2	7.56 ± 0.9	C_15_H_24_
63	γ-cuprenene	1541	1532 [18]	n.d.	n.d.	0.45 ± 0.07	C_15_H_28_O
64	(E)-nerolidol	1563	1561 [18]	0.47 ± 0.02	n.d.	0.86 ± 0.01	C_15_H_26_O
65	geranyl butanoate	1565	1562 [18]	0.48 ± 0.02	0.26 ± 0.01	0.47 ± 0.03	C_14_H_24_O_2_
66	jasmolactone, extra C	1574	1566 [18]	0.46 ± 0.06	n.d.	n.d.	C_11_H_18_O_2_
67	n-hexadecane	1595	1600 [16]	0.36 ± 0.01	n.d.	0.03 ± 0.05	C_16_H_34_
68	dodecyl acetate	1606	1607 [16]	n.d.	0.47 ± 0.01	n.d.	C_14_H_28_O_2_
69	1-hexadecene	1608	1588 [16]	0.45 ± 0.05	n.d.	0.28 ± 0.01	C_16_H_32_
70	eremophilone	1755	1756 [16]	n.d.	0.35 ± 0.04	0.55 ± 0.07	C_15_H_24_O_2_
71	not identified	1759		n.d.	0.69 ± 0.02	n.d.	--
	monoterpene hydrocarbons (%)			33.57 ± 1.2	6.84 ± 0.7	5.83 ± 0.6	
	sesquiterpene hydrocarbons (%)			24.64 ± 0.8	50.4 ± 1.2	51.920.2	
	oxygenated sesquiterpenes (%)			0.47 ± 0.02	1.04 ± 0.04	2.25 ± 0.2	
	oxygenated monoterpenes (%)			2.59 ± 0.6	32.7 ± 0.1	29.94 ± 0.3	
	others (%)			12.22 ± 0.1	8.56 ± 0.2	10.06 ± 0.1	
	total (%)			99.17	98.87	99.21	

LRI ^a^ = determined linear retention index; LRI ^b^ = Linear retention index from reference; $\bar{x}$ ± SD = percentage and standard deviation. Values are the average of three determinations. n.d. = not detectable; MF = molecular formula.

**Table 2:** Volatile chemical composition of *Lathyrus odoratus* flowers in polar column in different collection periods (2023).

HP-INNOWax							
N°	Compound	LRI ^a^	LRI ^b^	March (% ± SD)	May (% ± SD)	July (% ± SD)	MF
1	α-pinene	1072	1076 [19]	0.49 ± 0.22	n.d.	n.d.	C_10_H_18_
2	myrcene	1172	1174 [19]	0.48 ± 0.01	1.02 ± 0.03	2.07 ± 0.05	C_10_H_16_
3	limonene	1200	1203 [20]	0.32 ± 0.12	n.d.	n.d.	C_10_H_16_
4	(E)-β-ocimene	1256	1266 [20]	12.14 ± 1.82	7.21 ± 0.71	2.89 ± 0.32	C_10_H_16_
5	γ-terpinene	1257	1255 [20]	n.d.	0.58 ± 0.03	0.13 ± 0.01	C_6_H_10_O
6	pentanol	1261	1258 [21]	n.d.	0.2 ± 0.01	0.1 ± 0.01	C_5_H_12_O
7	p-cymene	1279	1280 [20]	n.d.	0.66 ± 0.05	0.10 ± 0.01	C_8_H1_8_O
8	acetoin	1301	1296 [22]	0.26 ± 0.04	0.16 ± 0.01	0.46 ± 0.02	C_4_H_8_O_2_
9	6 methyl-5-hepten-2-one	1319	1338 [23]	n.d.	0.74 ± 0.02	n.d.	C_8_H_14_O
10	1-hexanol	1330	1340 [24]	n.d.	0.36 ± 0.03	n.d.	C_6_H_14_O
11	(Z)-3-hexenol	1332	1348 [25]	n.d.	n.d.	1.25 ± 0.21	C_10_H_18_O_2_
12	cis-rose oxide	1340	1351 [26]	n.d.	n.d.	0.05 ±0.09	C_10_H_18_O
13	1,2,3-trimethyl benzene	1351	1355 [20]	n.d.	0.17 ± 0.04	n.d.	C_9_H_12_
14	(E)-2-hexen-1-ol	1358	1360 [27]	n.d.	n.d.	0.18 ± 0.06	C_6_H_12_O
15	not identified	1361		n.d.	0.19 ± 0.05	n.d.	--
16	cis-alloocimene	1370	1382 [20]	n.d.	n.d.	0.03 ± 0.05	C_16_H_12_O
17	α-pinene oxide	1385	1384 [20]	n.d.	n.d.	0.15 ± 0.01	C_10_H_16_O
18	trans-alloocimene	1400	1409 [20]	0.56 ± 0.01	0.49 ± 0.01	n.d.	C_10_H_16_
19	cis-linalool oxide, furanoid	1440	1450 [19]	1.78 ± 0.11	1.37 ± 0.02	n.d.	C_11_H_19_NO_3_
20	aromadendrene	1446	1443 [28]	0.31 ± 0.01	0.48 ± 0.01	0.19 ± 0.01	C_15_H_24_
21	α-cubebene	1460	1466 [19]	1.14 ± 0.03	0.83 ± 0.02	n.d.	C_15_H_24_
22	2-phenylethyl isovalerate	1461	1459 [28]	n.d.	n.d.	0.25 ± 0.02	C_13_H_18_O_2_
23	α-ylangene	1497	1493 [19]	n.d.	n.d.	0.03 ± 0.01	C_15_H_24_
24	pentadecane	1500	1500 [29]	0.21 ± 0.03	0.09 ± 0.03	n.d.	C_15_H_32_
25	benzaldehyde	1527	1541 [30]	n.d.	n.d.	0.29 ± 0.04	C_7_H_6_O
26	linalool	1551	1553 [19]	8.94 ± 0.73	9.38 ± 0.76	2.88 ± 0.38	C_10_H_18_O
27	α-trans-bergamotene	1561	1573 [19]	42.09 ± 1.82	43.48 ± 1.60	24.82 ± 1.39	C_15_H_24_
28	7-epi-sesquithujene	1569	1576 [31]	5.21 ± 0.82	4.3 ± 0.55	2.03 ± 0.32	C_15_H_24_
29	α-cis-bergamotene	1580	1584 [31]	0.92 ± 0.05	0.34 ± 0.02	n.d.	C_15_H_24_
30	hexadecane	1600	1600 [26]	0.86 ± 0.03	1.94 ± 0.15	0.37 ± 0.02	C_15_H_24_
31	(E)-β-farnesene	1611	1585 [18]	1.46 ± 0.04	0.91 ± 0.03	0.08 ± 0.01	C_15_H_24_
32	β-sesquiphellandrene	1668	1679 [28]	7.03 ± 0.84	6.53 ± 0.52	0.33 ± 0.14	C_15_H_24_
33	(Z)-β-farnesene	1673	1668 [32]	1.45 ± 0.09	0.99 ± 0.04	0.19 ± 0.01	C_15_H_24_
34	germacrene D	1703	1710 [33]	0.68 ± 0.01	0.23 ± 0.01	n.d.	C_15_H_24_
35	β-curcumene	1721	1737 [34]	0.53 ± 0.04	0.96 ± 0.12	n.d.	C_15_H_24_
36	methy decyl ketone	1723	1710 [35]	n.d.	n.d.	0.21 ± 0.01	C_12_H_24_O
37	geranial	1731	1732 [28]	n.d.	0.2 ± 0.35	3.25 **±** 0.45	C_10_H_16_O
38	geranyl acetate	1752	1730 [29]	0.21 ± 0.01	0.41 ± 0.01	1.38 ± 0.22	C_15_H_24_
39	(E,E)-α-farnesene	1764	1781 [17]	0.11 ± 0.03	n.d.	0.22 ± 0.01	C_14_H_24_O_2_
40	nerol	1797	1797 [11]	1.59 ± 0.03	0.53 ± 0.35	2.84 ± 0.15	C_10_H_18_O
41	p-mentha-1(7),5-dien-2-ol	1831	1823 [20]	n.d.	n.d.	0.78 ± 0.02	C_10_H_16_O
42	geraniol	1866	1857 [19]	0.71 ± 0.03	1.58 ± 0.05	1.34 ± 0.14	C_10_H_18_O
43	benzyl alcohol	1899	1880 [26]	n.d.	n.d.	0.24 ± 0.05	C_16_H_14_O_2_
44	phenyl ethyl alcohol	1910	1897 [20]	8.16 ± 1.0	1.76 ± 0.8	30.01 ± 0.8	C_8_H_10_O
45	(E)-nerolidol	2027	2050 [20]	1.43 ± 0.5	1.21 ± 0.7	5.30 ± 1.2	C_15_H_26_O
46	α-kaurene	2048	2056 [28]	n.d.	n.d.	1.61 ± 0.23	C_20_H_32_
47	not identified	2276		0.47 ± 0.02	0.66 ± 0.04	n.d.	--
48	tricosane	2292	2300 [28]	n.d.	n.d.	0.31 ± 0.03	C_23_H_48_
49	4-vinylphenol	2398	2417 [36]	n.d.	n.d.	0.50 ± 0.02	C_8_H_8_O
50	pentacosane	2498	2500 [28]	n.d.	n.d.	0.17 ± 0.01	C_25_H_52_
	monoterpene hydrocarbons (%)			13.11 ± 1.8	7.38 ± 0.7	4.96 ± 0.4	
	sesquiterpene hydrocarbons (%)			63.36 ± 1.8	61.97 ± 1.6	27.92 ± 0.3	
	oxygenated sesquiterpenes (%)			0.47 ± 0.5	2.28 ± 0.1	6.91 ± 1.2	
	oxygenated monoterpenes (%)			11.28 ± 0.7	12.09 ± 0.7	24.27 ± 0.5	
	alcohols (%)			n.d.	n.d.	30.01 ± 0.7	
	others (%)			10.69 ± 0.3	15.47 ± 0.5	3.12 ± 0.6	
	total (%)			98.91	99.19	97.19	

LRI ^a^ = determined linear retention index; LRI ^b^ = linear retention index from reference; $\bar{x}$ ± SD = percentage and standard deviation. Values are the average of three determinations. n.d. = not detectable; MF = molecular formula.

**Table 3:** Enantioselective analysis of some chiral terpenes from *Lathyrus odoratus* L. flowers using β-cyclodextrin column.

Component	LRI ^a^	LRI ^b^	Composition (%)	*e.e.* (%)
(*1R*)-(+)-α-pinene	1007	1008 [37]	100	
(*S*)-(−)-limonene	1065	1075 [37]	100	
(R)-(+)-linalool	1242	1247 [37]	48.02 ± 1.4	3.96
(L)-(−)-linalool	1247	1250 [37]	51.98 ± 2.1	
(R)-(+)-germacrene D	1484	1466 [38]	100	
(R)-(E)-nerolidol	1700		100	

LRI ^a^: determined linear retention index; LRI ^b^: linear retention index from reference; *e.e* = enantiomeric excess.

**Table 4:** PCA scores showing the main components of *Lathyrus odorarus* L. in different months of harvest using DB5-ms and HP-INNOWax columns.

DB5-ms		
Month	PC 1	PC 2
March	−20.053	−0.060594
May	10.338	−2.9013
July	9.7158	2.9619
HP-INNOwax		
March	−9.8961	4.0448
May	−14.054	−3.6023
July	23.95	−0.44254

## Data Availability

The original contributions presented in the study are included in the article. Further inquiries can be directed to the corresponding author.
